# Suppression of microglial Ccl2 reduces neuropathic pain associated with chronic spinal compression

**DOI:** 10.3389/fimmu.2023.1191188

**Published:** 2023-07-11

**Authors:** Quan Li, Zongde Yang, Kun Wang, Zhi Chen, Hongxing Shen

**Affiliations:** ^1^Department of Spine Surgery, Renji Hospital, School of Medicine, Shanghai Jiao Tong University, Shanghai, China; ^2^Department of Orthopedics, Renji Hospital, School of Medicine, Shanghai Jiao Tong University, Shanghai, China; ^3^Department of Spine Surgery, Changhai Hospital, The Second Military Medical University, Shanghai, China

**Keywords:** microglial Ccl2 regulates CPC-pain chronic spinal compression, microglia, Ccl2, pain, inflammation

## Abstract

**Introduction:**

Chronic spinal compression is a common complication of spinal cord injury (SCI), which can lead to spinal stenosis or herniated discs. The ensuing neuropathic pain is often associated with the activation of microglia. In this investigation, our objective was to explore whether modifying the levels of chemokine (C-C motif) ligand 2 (Ccl2) in microglia could alleviate neuropathic pain resulting from chronic spinal compression.

**Methods:**

We used a public database to look for major altered gene associated in a SCI model established in rats. We then employed adeno-associated virus (AAV) vectors, expressing siRNA for the identified significantly altered gene under a microglia-specific TMEM119 promoter. We also tested the impact of this treatment in microglia *in vivo* on the severity of chronic spinal compression and associated pain using a ttw mouse model for progressive spinal compression.

**Results:**

We identified chemokine (C-C motif) ligand 2 (Ccl2) as the primary gene altered in microglia within a rat SCI model, utilizing a public database. Microglial Ccl2 levels were then found to be significantly elevated in disc specimens from SCI patients diagnosed with chronic spinal compression and strongly correlated with the Thompson classification of the degeneration level and pain score. Depletion of Ccl2 in microglia-specific TMEM119 promoter were developed to transfect mouse microglia *in vitro*, resulting in a proinflammatory to anti-inflammatory phenotypic adaption. *In vivo* depletion of Ccl2 in microglia mitigated the severity of chronic spinal compression and related pain in ttw mice, likely due to significant changes in pain-associated cytokines and factors.

**Conclusion:**

Disc microglia expressing high levels of Ccl2 may contribute to chronic spinal compression and SCI-associated pain. Therapeutically targeting Ccl2 in microglia could offer a potential avenue for treating chronic spinal compression and SCI-associated pain.

## Introduction

Chronic spinal compression is a prevalent disease worldwide, affecting up to 20% of the adult population (1). The incidence of chronic spinal compression is more common in men and increases with age, leading to significant morbidity, decreased quality of life, and increased healthcare costs (1). The severity of symptoms depends on the location and degree of compression and can range from mild to severe. Common symptoms include back pain, neck pain, numbness or tingling in the arms or legs, weakness in the arms or legs, and difficulty walking (2). If left untreated, chronic spinal compression can result in permanent nerve damage and paralysis (3).

There are various causes of chronic spinal compression, including degenerative changes in the spine, such as herniated discs, bone spurs, or spinal stenosis; trauma, such as spinal cord injury or fractures; tumors or cancer that affect the spine; inflammatory conditions, such as rheumatoid arthritis or ankylosing spondylitis; infections or abscesses in the spinal cord or vertebrae; and congenital conditions, such as spinal cord defects or abnormalities in the spinal canal (4).

Neuropathic pain is a common consequence of chronic spinal compression, which can be caused by various mechanisms. The compression of the spinal cord can cause damage to the nerves and disrupt their normal function, leading to the release of inflammatory mediators and activation of glial cells (5). Moreover, chronic spinal compression can lead to changes in the expression of neurotransmitters and receptors involved in pain signaling (6). These changes can result in increased sensitivity to painful stimuli and the development of chronic pain.

One of the crucial mechanisms underlying the formation of neuropathic pain by chronic spinal compression involves the activation of glial cells, particularly microglia (7). Microglia are resident immune cells of the central nervous system that are activated in response to injury or damage to nervous tissue (8). In chronic spinal compression, microglia are present in and around the compressed area and contribute to the development and progression of the condition (9). Polarized microglia exhibit different phenotypes with different functions (10). The M1 phenotype is pro-inflammatory and contributes to tissue damage, while the M2 phenotype is anti-inflammatory and promotes tissue repair and regeneration (11). In chronic spinal compression, microglia are polarized towards the M1 phenotype, which leads to the production of pro-inflammatory cytokines and chemokines that contribute to the development and progression of the condition (12). Modulating the polarization of microglia towards the M2 phenotype has been shown to alleviate the severity of chronic spinal compression and associated neuropathic pain in animal models (13). Microglia also contribute to the amplification of pain signals and the establishment of a persistent pain state (14). Therefore, microglial activation is considered a potential target for the treatment of neuropathic pain resulting from chronic spinal compression (15). However, there is currently a deficiency in *in vivo* methods that specifically target microglia and a lack of knowledge on the specific target factors in microglia that regulate their phenotype and affect the pathogenesis and pain associated with chronic spinal compression (13). The current study aimed to address these questions.

## Materials and methods

### Protocol approval and animal work

Approval for this study was obtained from the Research and Animal Ethics Association at Renji Hospital. For human specimens, healthy disc controls were collected from deceased donors who had no history of spinal disease or injury and had died from non-spine-related causes, such as head trauma. Discs from SCI patients were selected from those diagnosed with chronic spinal compression after SCI. Written consent forms were obtained prior to including the cases in the study. For mouse study, the wildtype and ttw mice (16) (Animal Laboratory of the Academy of Medical Sciences, Beijing, China) received viruses at 8 weeks of age, and analyzed at 24 weeks of age when they exhibit histological spinal compression, disc degeneration and spinal stenosis. Male mice were used in 4 experimental groups of 5 each. Group 1, wildtype mice of comparable age and gender (wildtype); Group 2: ttw mice (ttw); Group 3: ttw mice that had received orthotopic injection of AAVs carrying pTMEM119-Scr at 8 weeks of age; Group 4: ttw mice that had received orthotopic injection of AAVs carrying pTMEM119-si-Ccl2 at 8 weeks of age.

### Von Frey filament test

The Von Frey filament test, also known as the hind paw withdrawal test, was performed in accordance with previously established methods (17). In summary, mice were placed in a designated testing chamber, allowing them to acclimate and move freely. Next, the experimenter gently applied pressure to one hind paw using a series of calibrated Von Frey microfilaments, increasing the force progressively. The test aimed to determine the minimum force required to elicit a clear, unambiguous withdrawal response of the hind leg. This withdrawal response, characterized by the mouse swiftly retracting its hind paw, is indicative of the sensitivity to the applied mechanical stimulus. The test was repeated multiple times, and the results were recorded and analyzed to assess the animals’ nociceptive thresholds.

### Griess assay

The Griess assay is a widely used protocol for the detection of nitric oxide (NO) production in biological samples. The assay is based on the reaction between NO and sulfanilamide, which forms a diazonium ion that then reacts with N-(1-naphthyl) ethylenediamine to produce a chromophore that can be detected by spectrophotometry. The general steps for the Griess assay include the collection and preparation of the biological sample, the addition of Griess reagent (a mixture of sulfanilamide and N-(1-naphthyl) ethylenediamine), and measurement of the absorbance of the resulting chromophore at a wavelength of 540 nm. The concentration of NO in the sample can then be determined by comparing the absorbance to a standard curve generated from known concentrations of a NO donor or NO-containing compound.

### Capsaicin behavior test

The evaluation of capsaicin behavior involved measuring the duration of licking behavior within a 5-minute period after injecting the hind paw of the mouse with capsaicin (3μg in 10μl). The capsaicin solution was prepared by dissolving it in a mixture of 5% ethanol, 5% Tween-80, and 90% saline, which was obtained from Sigma-Aldrich.

### Cells, plasmids and AAVs

A mouse microglia cell line (EOC2), a mouse macrophage cell line (J774a.1) and a mouse fibroblast cell line (3T3) were all purchased from American Type Culture Collection (ATCC, Rockville, MD, USA). EOC2 cells were maintained in Dulbecco’s Modified Eagle Medium (DMEM) containing 10% fetal bovine serum (FBS, Sigma-Aldrich, Beijing, China) and 1% penicillin-streptomycin (PS, Sigma-Aldrich) in a humidified atmosphere of 5% CO_2_ at 37°C. J774a.1 cells were cultured in high glucose DMEM containing 10% FBS and 1% PS in a humidified atmosphere of 5% CO_2_ at 37°C. 3T3 cells were grown in DMEM supplemented with 10% FBS, 2 mM L-glutamine (Sigma-Aldrich), 100 U/mL penicillin and 100 µg/mL streptomycin in a humidified atmosphere of 5% CO_2_ at 37°C. The culture medium was replaced every 2-3 days, and the cells were passaged when they reached approximately 80% confluence using 0.25% trypsin-EDTA (Sigma-Aldrich). The TMEM119 promoter was purchased from GeneCopoeia (#34837). The sequence for mouse si-Ccl2 was 5’-UUCUGAUCUUCUUCCAUCCTT-3’ and the scrambled sequence (Scr) was 5’-ACGUGACACGUUCGGAGAATT-3’. Transfection to generate adeno-associated viruses (AAVs) was performed using Lipofectamine 3000 reagent (Invitrogen) as per the manufacturer’s instructions. Human embryonic kidney 293 cells were transfected with prepared plasmids to generate AAV of serotype 6. Microglia were transduced *in vitro* using a multiplicity of infection (MOI) of 100. The orthotopic injection of AAVs was done with a single dose of 3x10^11^ viral particles in 100 µL total volume.

### DCF assay

The DCF assay was used for detecting the levels of reactive oxygen species (ROS) in microglia. To perform the assay, cells were first plated onto a 96-well plate, and then transfected with prepared plasmids. After 48 hours, the cells were incubated with a solution of 2’,7’-dichlorodihydrofluorescein diacetate (DCFH-DA), which was taken up by the cells and converted into a highly fluorescent product, dichlorofluorescein (DCF). Afterwards, phosphate-buffered saline (PBS) was used to wash away excess dye before measurement of the fluorescence intensity with a plate reader. The levels of ROS were quantified by comparing the fluorescence intensity of the experimental samples to that of control samples.

### Arginase activity

Measuring arginase activity in microglia involved a protocol that starts with cell lysis, followed by centrifugation to remove debris. The supernatant was then collected and mixed with a reaction buffer containing substrate (L-arginine). After incubation at 37°C, the reaction was stopped by the addition of 0.1M HCl, and the product (urea) was measured using a colorimetric assay. The activity of arginase was calculated by measuring the amount of urea produced over a specific time period, and normalizing it to the protein concentration of the sample.

### Flow cytometry

To perform flow cytometry analysis, the digested single cell fractions were incubated with a PE-conjugated TMEM119 antibody obtained from Becton-Dickinson Biosciences, located in Shanghai, China. To detect the expression of GFP, direct fluorescence using the FITC channel was used. The flow cytometry data was analyzed and presented with FlowJo software developed by Flowjo LLC, a company based in Ashland, Oregon, USA. The analysis included the determination of the expression levels of TMEM119 and GFP in the single cell fractions from the disc specimens of healthy donors and SCI patients and from experimental mice.

### Immunocytochemistry, H&E staining and ELISA

In cultured cells, GFP was detected by direct fluorescence. The mouse spine tissue was fixed with formalin and embedded in paraffin. Thin sections of the tissue were then cut using a microtome and placed onto glass slides. The sections are then deparaffinized and rehydrated using a series of alcohol solutions. The sections are then stained with hematoxylin, which stains the nuclei of the cells blue, and eosin, which stains the cytoplasm and extracellular matrix pink. The slides are then dehydrated again and coverslipped. Enzyme-linked immunosorbent assay (ELISA) was performed to measure the levels of mouse Ccl2 (ab208979; Abcam, Hangzhou, China), mouse Il1β (ab197742; Abcam), mouse tumor necrosis factor alpha (TNFα, ab208348; Abcam), mouse interferon gamma (IFNɣ, ab282874; Abcam), mouse Il6 (ab100712, Abcam), mouse Il17 (ab100702; Abcam), mouse arginase 1 (ARG1, ab269541; Abcam), mouse CD163 (ab272204, Abcam), mouse transforming growth factor β1 (TGFβ1, ab119557, Abcam) and mouse Il10 (ab108870, Abcam) in cell lysis. Specific kits were utilized as per the manufacturer’s instructions.

### Real-time quantitative polymerase chain reaction

Qiagen bioproducts were used for RNA extraction, cDNA synthesis and RT-qPCR experiments. Primers for mouse Ccl2: Forward: AGGTCCCTGTCATGCTTCTG, Reverse: AAGGCATCACAGTCCGAGTC. Primers for mouse GAPDH: Forward: ACTCCACTCACGGCAAATTC, Reverse: TCTCCATGGTGGTGAAGACA. Glyceraldehyde 3-phosphate dehydrogenase (GAPDH) was determined to be consistent among samples and was therefore utilized as a reference gene to standardize the expression levels of the genes under examination.

### Bioinformatics

To use GEO2R online tool for gene expression analysis, the first step was to select the dataset of our interest from the GEO database (GSE45006) and make multiple groups of samples for comparison. Then, the appropriate options for analysis were selected. The online tool performed statistical analysis and identified differentially expressed genes between the two or more groups and showed downloadable results. Next, for single-cell RNAseq data analysis, panglaodb.se was used. First, select the dataset of interest and choose the tissue type of interest (“spine” in this study). Then, search for genes of interest and visualize the expression patterns in different cell types using t-SNE co-layout.

### Statistical analysis

The statistical analyses were conducted using GraphPad Prism software (GraphPad Software, Inc., La Jolla, CA, USA). The analysis involved the use of one-way ANOVA with a Bonferroni correction. *Post-hoc* tests were applied for determining difference between two groups. The results were presented as mean ± standard deviation (SD), and p values less than 0.05 were considered statistically significant. Conversely, p values greater than 0.05 were considered as not statistically significant (ns).

## Results

### Microglia are the major sources for the upregulated Ccl2 after SCI

**Figure 1:**
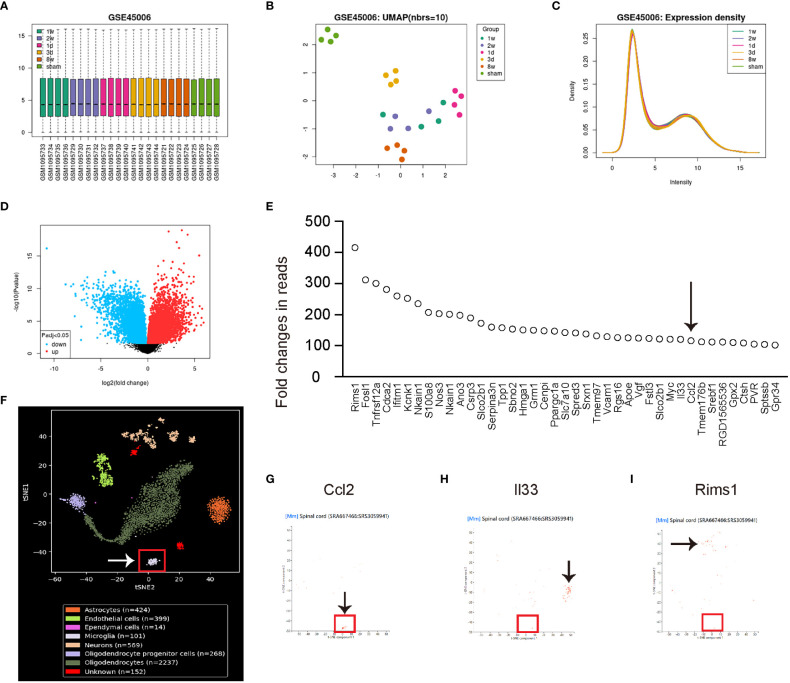
Microglia are the major sources for the upregulated Ccl2 after SCI. The quality control measurements taken to analyze altered genes associated with microglia following spinal cord injury (SCI). The data was obtained from the GEO database GSE45006. **(A–E)** GEO database GSE45006. **(A)** Principal component analysis (PCA). **(B)** Uniform Manifold Approximation and Projection (UMAP) plot. **(C)** Expression density analysis. **(D)** Volcano map displaying genes with significant differences, characterized by an adjusted p-value lower than 0.05 and a minimum 2-fold change. **(E)** List of highly altered genes after SCI with at least a 100-fold change. Arrow pointed to Ccl2. **(F–I)** Co-analysis with single-cell RNAseq data from SRA667466: SRS3059941. **(F)** tSNE layout of the cell clusters, noting that microglia were put in a red rectangle. **(G–I)** Co-analysis with Ccl2 **(G)**, interleukin 33 (Il33, **H**) and regulating synaptic membrane exocytosis 1 (Rims1, **I**). Arrows pointed to major cell clusters expressing co-analyzed genes.

Spinal cord injury (SCI) is a significant contributing factor to chronic spinal compression, as patients with SCI are at an increased risk of developing conditions such as spinal stenosis or herniated discs. To investigate the underlying mechanisms of chronic spinal compression caused by SCI, we searched for appropriate SCI models in public databases and identified a GEO database (GSE45006). In this research, a SCI model was developed in rats by employing an aneurysm clip impact-compression injury at the thoracic spinal cord (T7) level. Following the injury, RNA was extracted from the epicenter region of the injured spinal cord and analyzed using Affymetrix GeneChip arrays. Samples from sham-operated rats served as controls, and these were compared to SCI samples collected at various time points, including 1 day, 3 days, 1 week, 2 weeks, and 8 weeks post-injury. This comprehensive time-course analysis allowed for the examination of gene expression changes and their potential implications in the spinal cord injury response and recovery process. To identify the major altered genes associated with microglia, we first conducted a set of quality controls, including a principal component analysis (PCA) to reduce the dimensionality of the data by transforming the original variables into new ones linear with original variable combinations (**Figure 1A**), a Uniform Manifold Approximation and Projection (UMAP) plot to use machine learning technique for dimensionality reduction and data visualization (**Figure 1B**), and an expression density analysis as a graphical method to represent gene expression data for visualizing the distribution of gene expression values within multiple samples (**Figure 1C**). Our results demonstrated that the data were suitable for analysis (**Figures 1A-C**). We then used an adjusted p value lower than 0.05 and at least a 2-fold change to select the significantly differed genes, which were shown in a volcano map (**Figure 1D**). From the list of highly altered genes after SCI, we identified those with at least a 100-fold change (**Figure 1E**). Among these genes, we found that only Ccl2 was predominantly expressed in microglia with the aid of co-analysis with single-cell RNAseq data from SRA667466: SRS3059941 (**Figures 1F, G**). On the other hand, other highly altered genes such as interleukin 33 (Il33, **Figure 1H**) and regulating synaptic membrane exocytosis 1 (Rims1, **Figure 1I**) were mainly expressed by other cells. These findings suggest that microglia are the primary source of the upregulated Ccl2 following SCI.

### Microglial Ccl2 levels positively correlate with Thompson classification of the degeneration level and pain score in SCI patients

**Figure 2:**
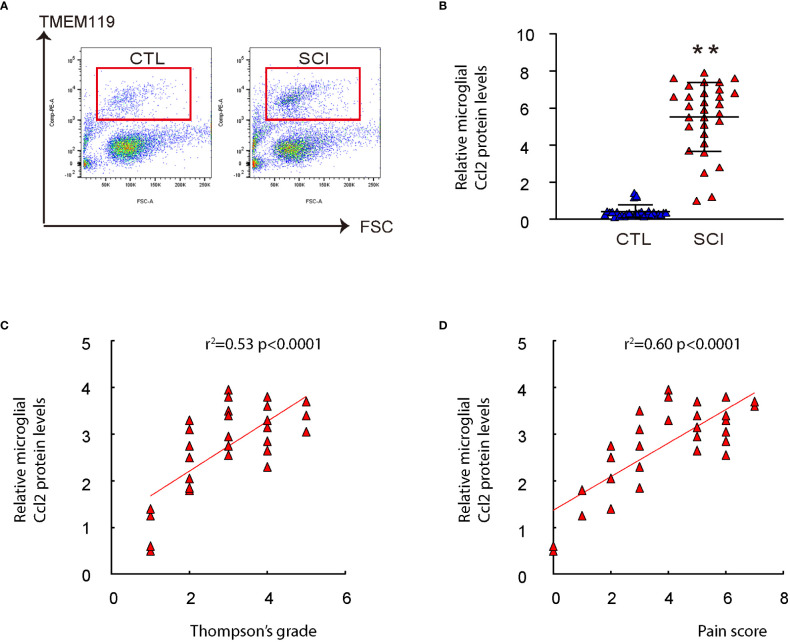
Microglial Ccl2 levels positively correlate with Thompson classification of the degeneration level and pain score in SCI patients. Disc specimens from 30 healthy donors (CTL) and 30 SCI patients diagnosed with chronic spinal compression were examined. **(A)** Microglia were isolated from the disc specimens by digesting them into a single-cell fraction and subjecting them to fluorescence-activated cell sorting (FACS) for TMEM119, a specific marker for microglia. Representative flow charts were shown. **(B)** ELISA for Ccl2 on isolated spinal microglia. **(C, D)** The correlation between microglial Ccl2 protein and Thompson classification of the degeneration level (C, ɣ^2 = ^0.53, p<0.0001) or pain score (D, ɣ^2 = ^0.60, p<0.0001) was assessed. **p<0.01.

To investigate the role of microglial Ccl2 in chronic spinal compression/SCI-associated pain, we examined disc specimens from 30 healthy donors (CTL) and 30 SCI patients diagnosed with chronic spinal compression (**Table 1**). We collected the Thompson classification of the degeneration levels and pain scores from the SCI patients and analyzed the disc specimens by digesting them into a single-cell fraction and subjecting them to fluorescence-activated cell sorting (FACS) for TMEM119, a specific marker for microglia (**Figure 2A**). By performing ELISA analysis for Ccl2, we found that the microglial Ccl2 levels in SCI patients were significantly higher compared to those from CTL (**Figure 2B**). Next, we assessed the correlation between microglial Ccl2 protein levels and the Thompson classification of the degeneration levels or pain scores in SCI patients, which showed a strong positive correlation in both (**Figures 2C, D**). Our findings suggest that disc microglia expressing high Ccl2 levels may play a role in chronic spinal compression/SCI-associated pain.

**Table 1:** Demographic details of Intervertebral disc donors.

Sample	Sex	Age (years)	Thompson’s grade	Pain Score	Relative disc Ccl2 mRNA
1	M	50	I	0	1.0
2	M	60	I	0	1.2
3	F	51	I	1	2.5
4	M	55	I	2	2.8
5	F	70	II	1	3.6
6	F	45	II	2	4.1
7	M	56	II	2	5.0
8	M	69	II	2	5.5
9	F	65	II	3	3.7
10	M	62	II	3	6.2
11	F	58	II	4	6.6
12	F	66	III	3	7.0
13	M	49	III	3	5.5
14	F	64	III	4	7.9
15	M	67	III	4	7.6
16	F	47	III	5	6.8
17	M	61	III	5	5.9
18	M	70	III	6	5.1
19	M	52	IV	3	4.6
20	F	68	IV	4	6.6
21	M	44	IV	5	5.3
22	F	57	IV	5	6.3
23	F	63	IV	6	6.6
24	M	71	IV	6	5.7
25	M	59	IV	6	7.6
26	F	46	IV	7	7.2
27	M	54	V	5	7.4
28	F	65	V	6	6.8
29	M	48	V	6	6.1
30	M	69	V	7	7.4

### Generation and validation of AAVs specifically targeting microglia and depleting Ccl2

**Figure 3:**
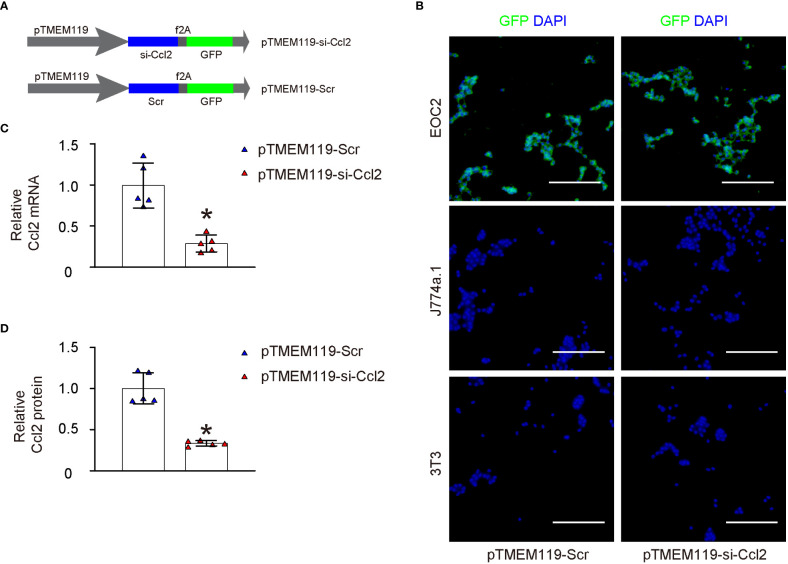
Generation and validation of AAVs specifically targeting microglia and depleting Ccl2. **(A)** Development of plasmids expressing either a siRNA for Ccl2 (si-Ccl2) or a control scrambled sequence (Scr) under a microglia-specific TMEM119 promoter (pTMEM119-si-Ccl2; pTMEM119-si-Scr). The plasmids also contained a GFP reporter co-controlled by the TMEM119 promoter through a f2A linker. **(B)** Both plasmids were used to transfect a mouse microglia cell line (EOC2), a mouse macrophage cell line (J774a.1), and a mouse fibroblast cell line (3T3). The specificity of the TMEM119 promoter for microglia was confirmed as only the transfected EOC2 cells appeared to be green fluorescent in culture. **(C, D)** RT-qPCR for Ccl2 mRNA **(C)** and ELISA for Ccl2 protein **(D)** levels. *p<0.05. Scale bars were 100µm.

In order to explore the possibility of decreasing Ccl2 levels in microglia and its impact on chronic spinal compression or SCI-related pain, we designed plasmids that expressed either a Ccl2-targeting siRNA (si-Ccl2) or a scrambled sequence (Scr) as a control. These plasmids were driven by a microglia-specific TMEM119 promoter. (pTMEM119-si-Ccl2; pTMEM119-si-Scr). The plasmids also contained a GFP reporter co-controlled by the TMEM119 promoter through a f2A linker (**Figure 3A**). Both plasmids were used to transfect a mouse microglia cell line (EOC2), a mouse macrophage cell line (J774a.1), and a mouse fibroblast cell line (3T3). The specificity of the TMEM119 promoter for microglia was confirmed as only the transfected EOC2 cells appeared to be green fluorescent in culture (**Figure 3B**). Furthermore, transfecting EOC2 cells with pTMEM119-si-Ccl2 significantly reduced Ccl2 mRNA (**Figure 3C**) and protein (**Figure 3D**) levels, confirming the efficacy of the pTMEM119-si-Ccl2 plasmid.

### Depletion of Ccl2 in microglia induces a proinflammatory to anti-inflammatory phenotypic adaption

**Figure 4:**
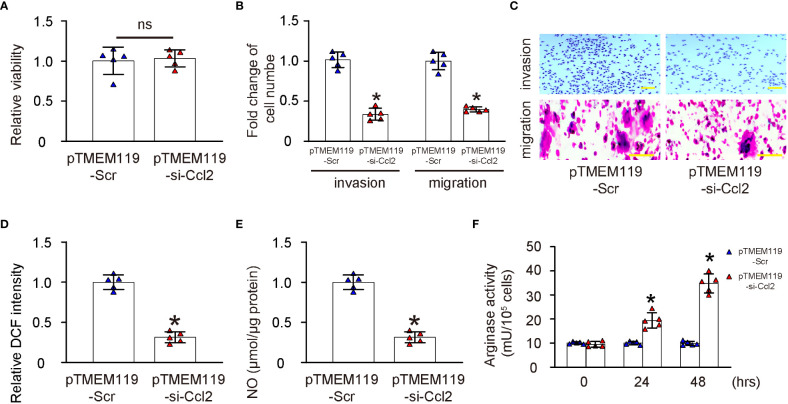
Depletion of Ccl2 in microglia induces a proinflammatory to anti-inflammatory phenotypic adaption. **(A)** Assessment of growth of microglia transfected with either pTMEM119-si-Ccl2 or pTMEM119-Scr by a CCK-8 assay. **(B, C)** Cell invasion assay and migration assay by quantification **(B)** and by representative images **(C, D)** DCF assay. **(E)** Griess assay. **(F)** Arginase activity. *p<0.05. ns, non-significant. Scale bars were 100µm.

To assess the effects of microglial Ccl2 on microglia properties, first we assessed the growth of microglia transfected with either pTMEM119-si-Ccl2 or pTMEM119-Scr by a CCK-8 assay. We found that depletion of Ccl2 in microglia did not significantly alter the growth or proliferation of microglia (**Figure 4A**). Next, the invasiveness and migratory potential of the transfected microglia were assessed, showing decreases in both by Ccl2 depletion (**Figures 4B, C**). ROS and NO are important properties of proinflammatory microglia, and were found both significantly reduced by a DCF assay (**Figure 4D**) and a Griess assay (**Figure 4E**), respectively. Arginase activity represents the anti-inflammatory function of microglia and showed significantly increased by Ccl2 depletion (**Figure 4F**). Together, these data suggest that depletion of Ccl2 in microglia reduces their invasion, migration and production of ROS and NOS, but increase their arginase activity, suggesting a proinflammatory to anti-inflammatory phenotypic adaption.

### Depletion of Ccl2 in microglia reduces chronic spinal compression-associated pain

**Figure 5:**
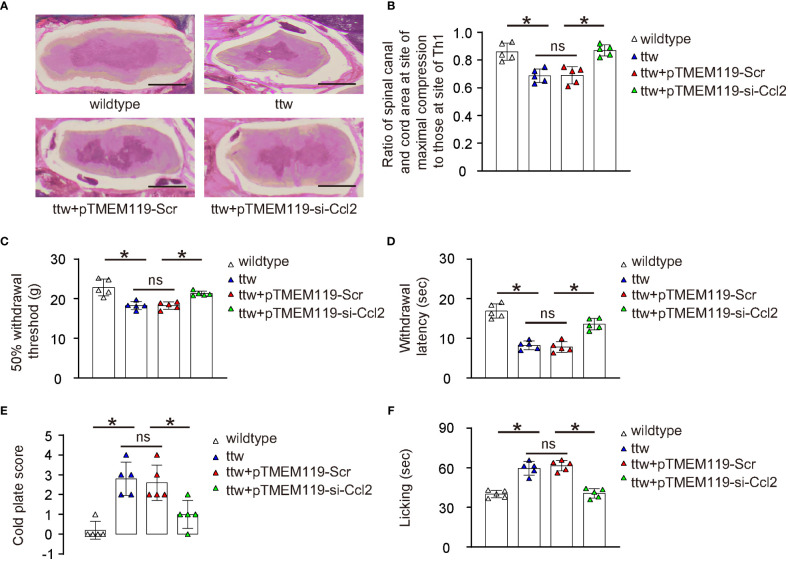
Depletion of Ccl2 in microglia reduces chronic spinal compression-associated pain. The effects of microglial depletion of Ccl2 on chronic spinal compression and its associated pain were then examined in a mouse model (ttw) *in vivo*. Four groups of mice were included in this experiment and all were analyzed at 24 weeks of age. Group 1, wildtype mice of comparable age and gender (wildtype); Group 2: ttw mice (ttw); Group 3: ttw mice that had received orthotopic injection of AAVs carrying pTMEM119-Scr at 8 weeks of age; Group 4: ttw mice that had received orthotopic injection of AAVs carrying pTMEM119-si-Ccl2 at 8 weeks of age. **(A)** H&E staining at analysis. **(B)** The degree of the sickness was quantified by the ratio of spinal canal and cord are at site of maximal compression (mostly C2-C3) to those at the Th1 site. **(C, D)** The mechanical **(C)** and thermal **(D)** pain was evaluated by a Von Frey filament test. **(E)** The cold plate score in a cold plate test. **(F)** The paw licking duration in a Capsaicin test. *p<0.05. ns, non-significant. Scale bars were 100µm.

The effects of microglial depletion of Ccl2 on chronic spinal compression and its associated pain were then examined in a mouse model (ttw) *in vivo*. The ttw mouse has a mutation in the type IX collagen gene that leads to the development of progressive spinal degeneration similar to that seen in humans with chronic spinal compression. The pTMEM119-si-Ccl2 or pTMEM119-Scr plasmids were packaged into AAVs serotype 6 to transduce mouse microglia *in vivo*. The experiment included a total of 4 groups of mice that were all analyzed at 24 weeks of age. Group 1, wildtype mice of comparable age and gender (wildtype); Group 2: ttw mice (ttw); Group 3: ttw mice that had received orthotopic injection of AAVs carrying pTMEM119-Scr at 8 weeks of age; Group 4: ttw mice that had received orthotopic injection of AAVs carrying pTMEM119-si-Ccl2 at 8 weeks of age. To assess the pathology of chronic spinal compression, H&E staining was performed (**Figure 5A**) and the degree of the sickness was quantified by the ratio of spinal canal and cord are at site of maximal compression (mostly C2-C3) to those at the Th1 site (**Figure 5B**). Our data showed significant spinal compression in ttw mice that were untreated or treated with control Scr AAVs, compared to wildtype mice (**Figures 5A, B**). However, the spinal compression was significantly attenuated in ttw mice treated with si-Ccl2 AAVs (**Figures 5A, B**). The pain was measured with different methods. First, the mechanical (**Figure 5C**) and thermal (**Figure 5D**) pain was evaluated by a Von Frey filament test, showing significant defect in ttw mice untreated or treated with control Scr AAVs, compared to wildtype mice (**Figures 5C, D**). However, both mechanical and thermal pain were significantly attenuated in ttw mice treated with si-Ccl2 AAVs (**Figures 5C, D**). Next, in a cold plate experiment to assess cold-related pain, the cold plate score was significantly higher in ttw mice untreated or treated with control Scr AAVs than wildtype mice (**Figure 5E**). However, the cold plate score was significantly reduced in ttw mice treated with si-Ccl2 AAVs (**Figure 5E**). Finally, a Capsaicin test was performed to measure chemical-related pain, which showed that the paw licking duration was significantly longer in ttw mice untreated or treated with control Scr AAVs than wildtype mice (**Figure 5F**). However, the paw licking duration was significantly reduced in ttw mice treated with si-Ccl2 AAVs (**Figure 5F**). Together, these data suggest that depletion of Ccl2 in microglia reduces severity of chronic spinal compression and its associated pain.

### Microglial Ccl2 levels in ttw mice are significantly reduced by si-Ccl2 virus

**Figure 6:**
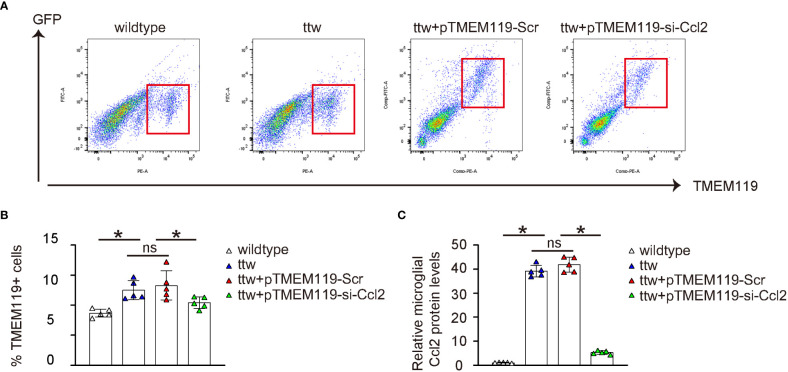
Microglial Ccl2 levels in ttw mice are significantly reduced by si-Ccl2 virus. **(A)** The spinal microglia were obtained from the mice using FACS based on TMEM119, as shown by representative flow charts. **(B)** Quantification of TMEM119+ microglia percentage. **(C)** ELISA for microglial Ccl2 levels. *p<0.05. ns, non-significant.

The spinal microglia were obtained from the mice using FACS based on TMEM119, as shown in **Figure 6A**. The results revealed significantly higher numbers of microglia from ttw mice untreated or treated with control Scr AAVs than in wildtype mice (**Figure 6B**). Conversely, the number of microglia was significantly decreased in ttw mice treated with si-Ccl2 AAVs (**Figure 6B**). To evaluate the levels of microglial Ccl2 protein, ELISA was used. Our results showed that Ccl2 levels were significantly higher in microglia from ttw mice untreated or treated with control Scr AAVs than in wildtype mice (**Figure 6C**). However, ttw mice treated with si-Ccl2 AAVs showed significantly reduced levels of Ccl2 in microglia (**Figure 6C**). Therefore, microglial Ccl2 levels in ttw mice are significantly reduced by si-Ccl2 virus.

### Depletion of Ccl2 in microglia alters pain-associated cytokines and factors in spine

**Figure 7:**
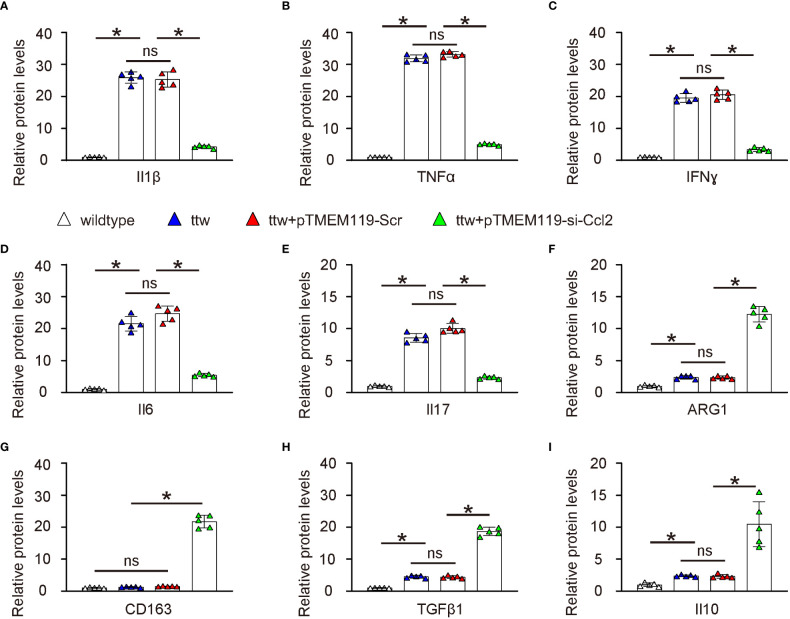
Depletion of Ccl2 in microglia alters pain-associated cytokines and factors in spine ELISA for known cytokines and factors related to pain. **(A)** Il1β. **(B)** TNFα. **(C)** IFNɣ. **(D)** Il6. **(E)** Il17. **(F)** ARG1. **(G)** CD163. **(H)** TGFβ1. **(I)** Il10. *p<0.05. ns, non-significant.

Finally, we examined the underlying mechanisms of the reduced pain in ttw by microglial Ccl2 depletion. We analyzed known cytokines and factors related to pain. Interestingly, we found that all factors that induce pain such as Il1β (**Figure 7A**), TNFα (**Figure 7B**), IFNɣ (**Figure 7C**), Il6 (**Figure 7D**) and Il17 (**Figure 7E**) were all significantly reduced by microglial Ccl2 depletion, while all factors that reduce pain such as ARG1 (**Figure 7F**), CD163 (**Figure 7G**), TGFβ1 (**Figure 7H**) and Il10 (**Figure 7I**) were all significantly induced by microglial Ccl2 depletion.

## Discussion

Chronic spinal compression resulting from SCI can cause spinal stenosis or herniated discs (18). In this study, Ccl2 was identified as the major altered gene associated with microglia in an SCI rat model, and elevated levels of microglial Ccl2 were found in disc specimens from SCI patients with chronic spinal compression. *In vitro* experiments showed that depletion of Ccl2 in microglia resulted in a proinflammatory to anti-inflammatory phenotypic adaptation. Depletion of Ccl2 in microglia also reduced severity of chronic spinal compression and its associated pain in a ttw mouse model, which was confirmed by changes in pain-associated cytokines and factors in the spine. These findings suggest that Ccl2 in microglia may be a potential therapeutic target for treating chronic spinal compression/SCI-associated pain.

Previous studies have shown that microglia activation contributes to chronic pain after SCI (19), but the underlying mechanisms and the key factor important for microglial phenotypic adaptation during chronic spinal compression have not been fully understood (20). Our study provides new insight into the role of microglial Ccl2 in chronic spinal compression/SCI-associated pain and suggests that Ccl2 in microglia may be a potential therapeutic target.

CCL2 is known as a chemokine that plays a crucial role in the recruitment and activation of macrophages/microglia to sites of inflammation. The absence of CCL2 may inhibit the NF-κB pathway, which is a key regulator of proinflammatory responses, leading to reduced production of proinflammatory cytokines and chemokines to create an environment that promotes the polarization of macrophages/microglia towards an anti-inflammatory phenotype (21). In addition, CCL2 knockout in macrophages/microglia may promote STAT6 activation, which in turn increases anti-inflammatory cytokine production, such as IL-10, and the expression of anti-inflammatory surface markers, such as CD206 and Arg1 (22, 23). Furthermore, CCL2 knockout may enhance the activation of the peroxisome proliferator-activated receptor gamma (PPARγ) pathway, which promotes the anti-inflammatory phenotype by suppressing proinflammatory gene expression and inducing the expression of genes associated with tissue repair and resolution of inflammation (24, 25). Lastly, the absence of CCL2 may alter the balance of proinflammatory and anti-inflammatory macrophages/microglia in the tissue, leading to reduced infiltration of proinflammatory macrophages/microglia and allowing for a shift towards an anti-inflammatory population (26, 27). Overall, the knockout of CCL2 in macrophages/microglia can promote the proinflammatory to anti-inflammatory transition through the modulation of various signaling pathways, ultimately promoting an anti-inflammatory environment.

IL17, which is known to be generated from Th17 cells, was found significantly decreased in the microglial Ccl2-knockout group compared to the controls. The interplay between microglial cells and Th17 cells is crucial for the immune response in the central nervous system (28). Microglia secrete chemokines and cytokines, such as CCL2, which promote the recruitment and survival of Th17 cells, a subset of CD4+ T helper cells producing the proinflammatory cytokine IL-17 (29). In microglial CCL2 knockout models, the reduced expression of CCL2 may lead to impaired recruitment of Th17 cells and decreased IL-17 production, potentially attenuating the overall inflammatory response and promoting a shift towards a more anti-inflammatory environment (30). The complex and multifaceted relationship between microglial cells and Th17 cells influences each other’s function in the context of central nervous system inflammation.

However, this study has some limitations. Firstly, although SCI is a major cause or contributing factor to chronic spinal compression, the condition can also occur in individuals without a history of SCI (31). Thus, while SCI is a crucial factor in the development of chronic spinal compression, it is not the sole cause of the condition (31). Secondly, the use of a single mouse model may restrict the generalizability of the findings. It would be beneficial to replicate the results in other mouse models, larger animal models, and investigate the effects of Ccl2 depletion on other aspects of SCI, such as inflammation and neuronal damage (32). Although the ttw mouse model used in this study is a valuable tool for investigating chronic spinal compression, it is a genetic model and may not fully replicate the etiology of chronic spinal compression caused by SCI in humans (16). Moreover, the spinal degeneration in ttw mice is progressive and may not accurately represent the acute phase of SCI that leads to chronic spinal compression (16). Additionally, the ttw model may not fully capture the complexity of human spinal cord injury, including other factors contributing to the development of chronic spinal compression, such as inflammation and tissue remodeling (16). Other models that are commonly used for studying chronic spinal compression include SCI models induced by contusion, hemisection, or transection, and models of disc herniation induced by puncture or needle compression (33). These models have their own advantages and disadvantages, and the choice of model often depends on the specific research question being addressed (33). Moreover, this study only examined the effects of Ccl2 depletion in microglia, and other cells in the spine may also contribute to chronic spinal compression/SCI-associated pain (34). Future studies could investigate the effects of Ccl2 depletion in other cells in the spine and in other cell types in the nervous system, specifically focusing on the crosstalk among different cell types in the microenvironment using Ccl2 as a mediator (35). Combining these models could strengthen the power and generalizability of this study. Finally, while this study’s primary focus was on the role of microglial Ccl2 in chronic spinal compression and associated pain, the potential implications of our findings may extend to a variety of other neurological conditions. Alzheimer’s disease, for instance, is characterized by the accumulation of amyloid-beta plaques, which are known to activate microglia and induce a proinflammatory state (36). It is plausible that modulating the levels of Ccl2 in microglia could alter this inflammatory response, potentially slowing the progression of the disease (36). Similarly, in multiple sclerosis, a condition marked by chronic inflammation and demyelination, Ccl2-mediated microglial activity could be contributing to the disease pathogenesis (37). It would be of interest to explore if reducing Ccl2 levels could ameliorate the inflammatory and degenerative aspects of this condition (37). Lastly, the role of microglia and inflammation is increasingly recognized in Parkinson’s disease, where dopaminergic neuron loss occurs. Altering Ccl2 activity might impact microglial responses and, consequently, neuronal survival in this context (38). However, each of these diseases has a distinct pathological process, and the effect of Ccl2 modulation could vary accordingly. Further research is required to delineate these possibilities and uncover the full therapeutic potential of targeting Ccl2 in microglia.

In summary, our study highlights the significance of microglial Ccl2 in chronic spinal compression/SCI-associated pain. The findings suggest that targeting Ccl2 expression in microglia may be a promising therapeutic approach. While the use of AAVs for gene therapy *in vivo* has shown promise, it is important to acknowledge the potential limitations and risks associated with this approach (39). Long-term depletion of Ccl2 in microglia may lead to unforeseen consequences, as these cells are involved in various physiological and pathological processes (40, 41). Furthermore, translating this research to clinical applications presents numerous challenges, including safety concerns, dosage optimization, and potential off-target effects (39). To address these issues, additional preclinical studies are required to comprehensively evaluate the safety and efficacy of targeting Ccl2 in microglia and to better understand the long-term implications of this therapeutic strategy.

## Data availability statement

The original contributions presented in the study are included in the article/supplementary material. Further inquiries can be directed to the corresponding authors.

## Ethics statement

The studies involving human participants were reviewed and approved by Renji Hospital. The patients/participants provided their written informed consent to participate in this study. The animal study was reviewed and approved by Renji Hospital.

## Author contributions

QL, ZY, KW, ZC and HS contributed to study design, data collection and analysis. LQ did bioinformatics and wrote the manuscript. QL, ZY, KW, ZC and HS approved the manuscript to be published. HS is the guarantee of the study. All authors contributed to the article and approved the submitted version.
